# Identification of *N*-glycans with GalNAc-containing antennae from recombinant HIV trimers by ion mobility and negative ion fragmentation

**DOI:** 10.1007/s00216-021-03477-3

**Published:** 2021-07-30

**Authors:** David J. Harvey, Anna-Janina Behrens, Max Crispin, Weston B. Struwe

**Affiliations:** 1 Oxford Glycobiology Institute, Department of Biochemistry, University of Oxford, South Parks Road, Oxford, OX1 3QU UK; 2 Target Discovery Institute, Nuffield Department of Medicine, University of Oxford, Roosevelt Drive, Oxford, OX3 7FZ UK; 3 Present Address: GlycoEra AG, Grabenstrasse 3, 8952 Schlieren, Switzerland; 4 School of Biological Sciences, Faculty of Natural and Environmental Sciences, University of Southampton, Highfield Campus, Southampton, SO17 1BJ UK; 5 Chemistry Research Laboratory, Department of Chemistry, University of Oxford, South Parks Road, Oxford, OX1 3TA UK

**Keywords:** Negative ion fragmentation, Ion mobility, *N*-Glycans, *N*-Acetylgalactosamine, Human immunodeficiency virus

## Abstract

Negative ion collision-induced dissociation (CID) of underivatized *N*-glycans has proved to be a simple, yet powerful method for their structural determination. Recently, we have identified a series of such structures with GalNAc rather than the more common galactose capping the antennae of hybrid and complex glycans. As part of a series of publications describing the negative ion fragmentation of different types of *N*-glycan, this paper describes their CID spectra and estimated nitrogen cross sections recorded by travelling wave ion mobility mass spectrometry (TWIMS). Most of the glycans were derived from the recombinant glycoproteins gp120 and gp41 from the human immunodeficiency virus (HIV), recombinantly derived from human embryonic kidney (HEK 293T) cells. Twenty-six GalNAc-capped hybrid and complex *N*-glycans were identified by a combination of TWIMS, negative ion CID, and exoglycosidase digestions. They were present as the neutral glycans and their sulfated and α2→3-linked sialylated analogues. Overall, negative ion fragmentation of glycans generates fingerprints that reveal their structural identity.

## Introduction

Fragmentation by collision-induced dissociation of negative ions from *N*-linked glycans (those attached to proteins at asparagine in an Asn-Aaa-Ser(Thr) consensus sequence, where Aaa is any amino acid except proline) has been shown to provide a wealth of diagnostic cross-ring fragments. Each individual glycan spectrum is sufficient for determining most structural features without the use of derivatization such as permethylation or reducing end tagging [1]. Specifically, for non-acidic glycans, the method provides the composition of the glycan in terms of its constituent isobaric and isomeric monosaccharide composition, its topology in terms of antenna branching (which type of triantennary glycan is present, for example), the location of fucose residues, and the presence or absence of bisecting GlcNAc. Some of these structural features are difficult to determine by traditional methods such as exoglycosidase sequencing as these enzymatic reactions are often incomplete plus they are often time-consuming and expensive. For acidic glycans, those with sialic acid can be converted to neutral compounds by methyl ester [2] or amide formation and, depending on the method used, the resulting spectra provide information on sialic acid linkage by utilizing their different reactivities to the derivatizing reagents such that the linkage is revealed simply by mass difference [3, 4]. We have been systematically examining the negative ion fragmentation of different types of *N*-glycans [5–11] and in this report present negative ion collision-induced dissociation (CID) spectra and ion mobility properties of glycans containing *N*-acetylgalactosamine (GalNAc) terminating their antennae and their identification in the human immunodeficiency virus (HIV) envelope glycoprotein (Env) BG505 SOSIP.664 trimers expressed in HEK 293T cells.

The soluble, recombinant BG505 SOSIP.664 trimer is the prototype of a class of native-like, Env spike-mimetic immunogens that is now being pursued in various vaccine-development programs [12, 13]. In a paper from this laboratory [14], a quantitative site-specific *N*-glycosylation analysis identified the *N*-glycans attached to twenty of the twenty-six sites of the SOSIP.664 Env trimer. Among the glycans were a number of hybrid and complex species with GalNAc rather than the more normal galactose terminating the antennae. Here, we examine the fragmentation behaviour of these *N*-glycan structures. Although *N*-glycans whose antennae terminate in GalNAc are common in glycoproteins from some sources such as the human pituitary gland [15, 16], tenascin R [17], glycodelin [18], porcine cardiac tissue [19], glycoproteins expressed in HEK 293 (human protein C [20] and, membrane protein CD9P-1 [21]), HEK 293F cells (L-selectin [22], the receptor binding domain of the SARS-CoV-2 spike glycoprotein [23]), and some non-mammalian organisms [24], we have not found them in gp120 or gp41 expressed in HEK 293 cells until now. The relevant transferase has been detected in these cells by Do et al. [25] and exoglycosidase digestion confirmed the presence of GalNAc in the current samples. Understanding the range of glycan structures on recombinant glycoproteins, such as viral glycoproteins, is important in the characterization of candidate immunogens and for the interpretation of the resulting immune response. Here, we probe their fragmentation properties under negative ion CID and report their nitrogen cross sections recorded by travelling wave ion mobility mass spectrometry (TWIMS).

## Materials and methods

### Materials

Glycans were released from BG505 SOSIP.664 trimers that were transiently expressed in HEK 293T cells (Thermo Fisher Scientific, Waltham, MA) and purified by 2G12-affinity chromatography followed by size-exclusion chromatography, as previously described [26, 27]. The Env trimers were dissociated into their gp120 and gp41subunits in SDS-PAGE gels in the presence of dithiothreitol (DTT) and stained with Coomassie blue. The gp41 subunit lacks the transmembrane region and cytoplasmic tail and is referred herein as gp41_ECTO_ (i.e. ectodomain). Following destaining, the bands corresponding to gp120 and gp41_ECTO_ were excised and washed five times alternatingly with acetonitrile and water. The *N*-glycans were released in situ [28] with peptide *N*-glycosidase F (PNGase F, New England Biolabs, Ipswich MA), at 37 °C for 16 h, according to the manufacturer’s instructions. The released glycans were extracted from the gel by washing with water and dried in a SpeedVac concentrator. Reference glycans were obtained from Dextra Laboratories (Reading, UK).

Human pituitary follicle-stimulating hormone (hFSH^24/21^) was obtained from Dr. G. Bousfield (Wichita State University, KS). The *N*-glycans were released with PNGase F from reduced and carboxymethylated of purified hormone (13–30 μg) as described earlier [16] and desialylated by incubation with sialidase from *Arthrobacter ureafaciens*.

### Exoglycosidase digestions

Approximately half of the samples were examined directly by ESI-MS, and the remainder were desialylated with the non-specific neuraminidase from *Clostridium perfringens* (New England Bioscience) according to the manufacturer’s instructions. The nature of the monosaccharide constituents was confirmed by exoglycosidase digestions as reported earlier, e.g. [29].

### Sample preparation for mass spectrometry

Following release from the glycoproteins, all glycan samples were cleaned with a Nafion® 117 membrane as described earlier by Börnsen et al. [30] before examination by mass spectrometry. They were dissolved in a solution of methanol:water (1:1, v:v) containing ammonium phosphate (0.05 M, to maximize formation of [M+H_2_PO_4_]^−^ ions, the most common type of ion normally seen from biological samples). Formation of adducts, such as those from phosphate, gives stable ions and prevents extensive in-source fragmentation commonly seen with deprotonated ions. Samples were then centrifuged at 10,000 rpm (9503×*g*) for 1 min to sediment any particulate matter.

### Ion mobility mass spectrometers

Travelling wave ion mobility mass spectrometry experiments were carried out with a Synapt G2Si travelling wave ion mobility mass spectrometer (Waters, Manchester, UK) [31] fitted with a nano-electrospray (nESI) ion source. Thin-wall nanospray capillaries were used for introducing the samples (Waters, Manchester, UK). Ion source conditions were as follows: ESI capillary voltage, 1.0–1.2 kV cone voltage, 100–180 V, ion source temperature 80 °C. The T-wave velocity and peak height voltages were 450 m/s and 40 V respectively unless otherwise specified. Fragmentation was performed after mobility separation in the transfer cell, with argon as the collision gas. The collision cell voltage (60–130 V) was adjusted according to the parent ion mass to give an even distribution of fragment ions across the mass range. The instrument was externally mass calibrated with dextran oligomers (Glc_2-13_) from *Leuconostoc mesenteroides* and the same material was used to calibrate the drift cell [32, 33]. Data acquisition and processing were carried out using the Waters DriftScope (version 2.8) software and MassLynx™ (version 4.1). Estimated collisional cross sections (CCS), in nitrogen, were determined with the cross section calculation algorithm supplied with DriftScope or, in the case of calculations made from extracted fragment arrival time distributions (ATDs), by the method reported by Thalassinos et al. [34]. In these latter measurements, it was found that the drift times of the ions measured in the MS and CID spectra were slightly different (approx. 0.46 ms) necessitating a correction to be made [35, 36]. The scheme devised by Domon and Costello [37] was used to name the fragment ions. Interpretation of the fragmentation data followed the rules established earlier [5–7, 38, 39].

Spectra from the FSH sample were recorded with a Waters quadrupole time-of-flight (Q TOF) Ultima Global instrument. Samples (about 50 pmoles/μl) in 1:1 (v:v) methanol:water were infused with Proxeon (Proxeon Biosystems, Odense, Denmark) borosilicate capillaries at about 5 μl/min. The ion source was maintained at 120 °C, the infusion emitter potential was 1.1 kV, the cone was at 100 V, and the RF-1 voltage was set at 180 V. Spectra (2 s scans) were acquired with a digitization rate of 4 GHz and accumulated until a satisfactory signal to noise ratio had been obtained. For CID data acquisition, the parent ion was selected at low resolution (about 5 *m*/*z* mass window) to allow transmission of isotope peaks and fragmented with argon as collision gas at a pressure (recorded on the instrument’s pressure gauge) of 0.5 mBar. The voltage on the collision cell was adjusted with mass and charge to give an even distribution of fragment ions across the mass scale. Typical values were 80–120 V. Other voltages were as recommended by the manufacturer. The instrument was externally calibrated with *N*-glycans released from bovine fetuin and are cited to one decimal place. Data acquisition and processing were performed with MassLynx 4.1 as earlier.

## Results and discussion

### Ion mobility extraction of *N*-glycan spectra from the HIV samples

**Fig. 1:**
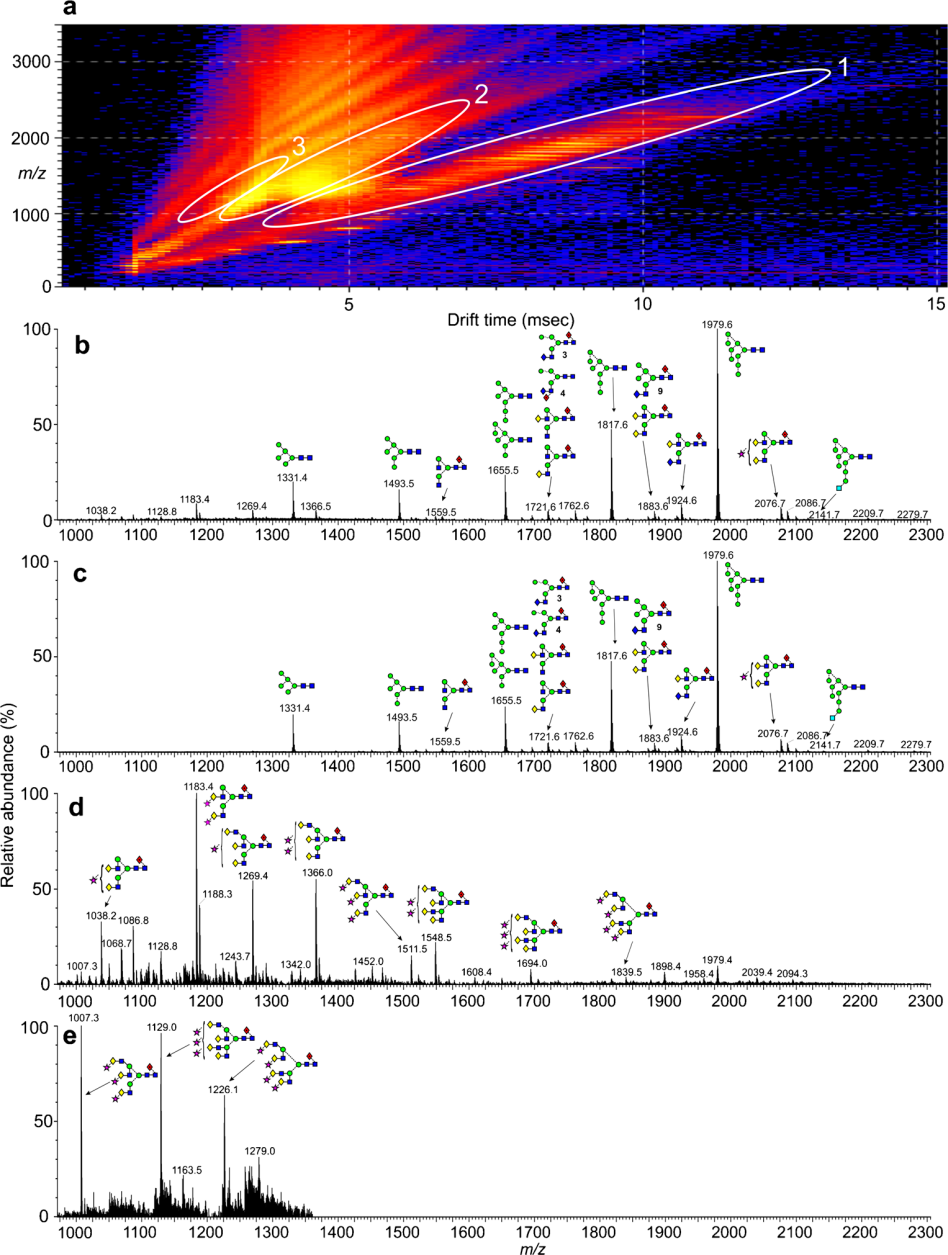
(**a**) DriftScope display of *N*-glycans released from gp120. (**b**) Negative ion ESI spectrum of the *N*-glycans. (**c**) Mobility-extracted singly charged ions from the spectrum shown in panel **b**. (**d**) Mobility-extracted doubly charged ions from the spectrum shown in panel **b**. (**e**) Mobility-extracted triply charged ions from the spectrum shown in panel **b**. Numbers in bold accompanying the structures are those of the glycans listed in Table 1. Symbols used for the glycans are  = mannose,  = GlcNAc,  = glucose,  = fucose,  = galactose,  = GalNAc, and  = Neu5Ac (sialic acid). Solid lines connecting the symbols are β-linkages; broken lines are α-linkages. The angle of the lines shows the linkage position (for more information, see [40])

**Fig. 2:**
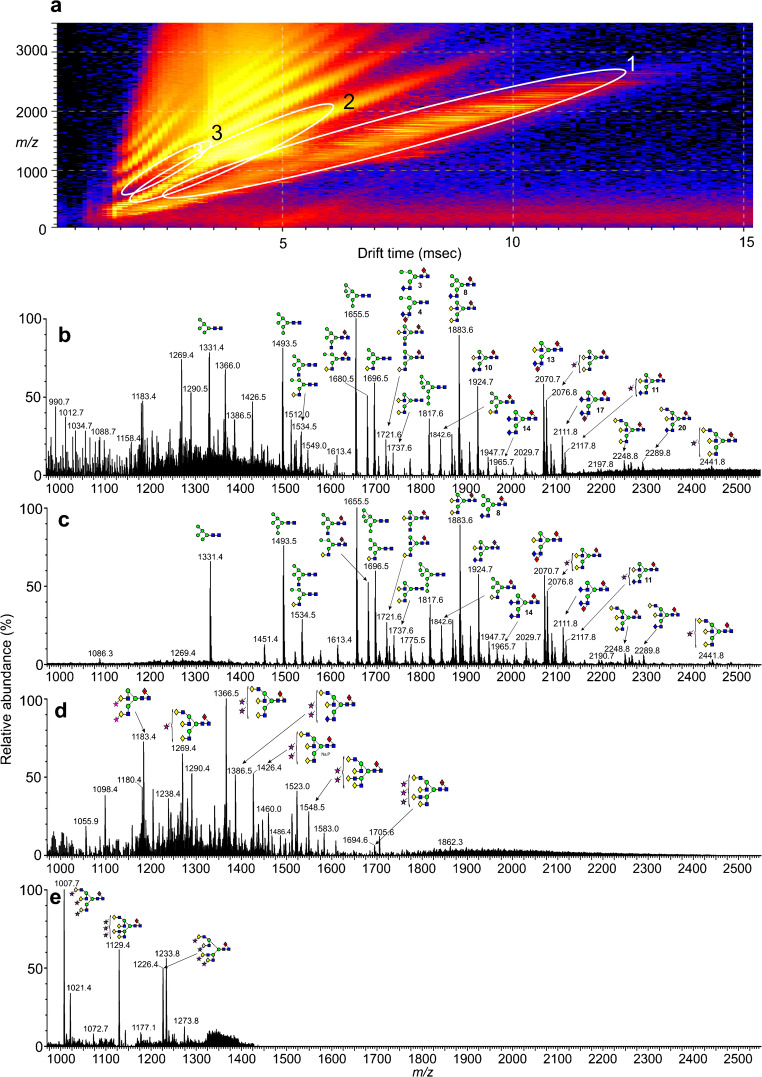
(**a**) DriftScope display of *N*-glycans released from gp41. (**b**) Negative ion ESI spectrum of *N*-glycans released from gp41. (**c**) Mobility-extracted singly charged ions from the spectrum shown in panel **b**. (**d**) Mobility-extracted doubly charged ions from the spectrum shown in panel **b**. (**e**) Mobility-extracted triply charged ions from the spectrum shown in panel **b**. Numbers in bold accompanying the structures are those of the glycans listed in Table 1

The ESI spectrum of the glycans released from gp120 gave a spectrum (Fig. 1b), similar to that obtained earlier [41] from a related sample, although the relative proportions of the glycans differed considerably. Ion mobility allowed the singly (Fig. 1c), doubly (Fig. 1d), and triply charged ions (Fig. 1e) to be separated, aiding spectral interpretation. The ESI spectrum of the glycans released from gp41 (Fig. 2b) was weaker but, again, ion mobility allowed clear spectra of the singly (Fig. 2c), doubly (Fig. 2d), and triply charged ions (Fig. 2e) to be extracted. In these spectra, neutral glycans produced [M+H_2_PO_4_]^−^ ions whereas the singly charged sialylated and sulfated glycans were detected as [M-H]^−^ species. Doubly and triply charged ions were present mainly as [M-H_2_]^2−^ and [M-H_3_]^3−^ species respectively although some sialylated glycans produced [M-H+Na+(H_2_PO_4_)]^2−^ ions. All identified GalNAc-containing glycans, with their respective ions, are listed in Table 1. A full list of the glycans present in these samples has been published in the Supplementary Information section of Behrens et al. 2016 [14].

**Table 1:**
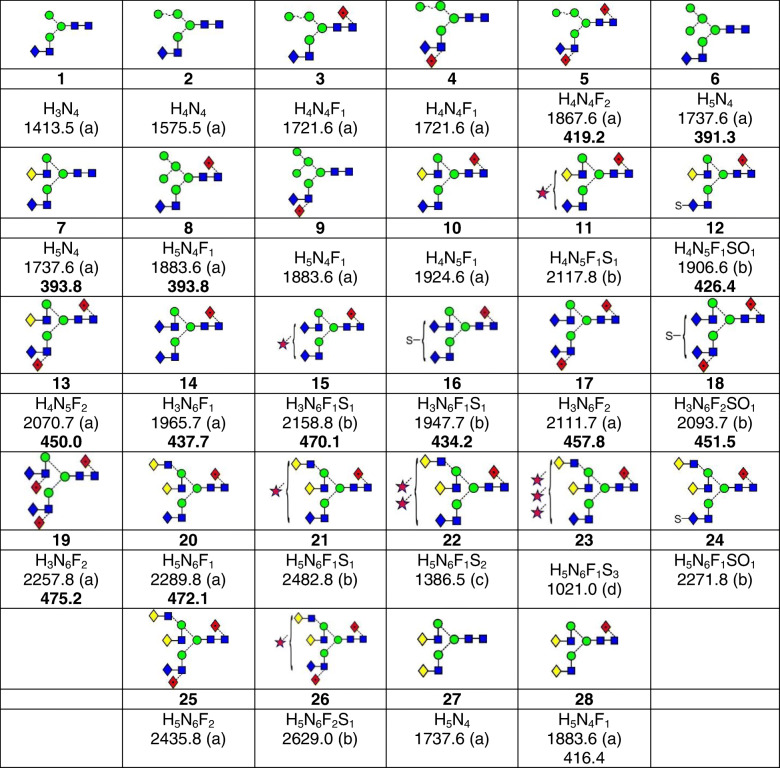
Structures and properties of the GalNAc-containing *N*-glycans. *N*, *N*-acetylhexosamine (i.e. HexNAc); *H*, hexose; *F*, fucose; *S*, *N*-acetylneuraminic acid (i.e. Neu5Ac); *SO*, sulfate. Shown below the composition are the monoisotopic molecular weight, the detected ion type in parentheses, and the estimated nitrogen cross section in bold where the ion abundance was sufficiently high to give a Gaussian ATD

(a) [M+H_2_PO_4_]^−^, (b) [M-H]^−^, (c) [M-H_2_]^2−^, (d) [M-H_3_]^3−^

### CID spectra

The general features of the negative ion fragmentation of high-mannose and complex *N*-glycans whose antennae terminate in galactose have been reported before [1, 5–7, 39] and will not be repeated here. Suffice it to say that features such as the branching pattern, linkage of sialic acid, the position of fucose substituents, and the presence or absence of bisecting GlcNAc residues are easily determined from the spectra as will be seen from the following discussion.

### Complex glycans containing GalNAc

**Fig. 3:**
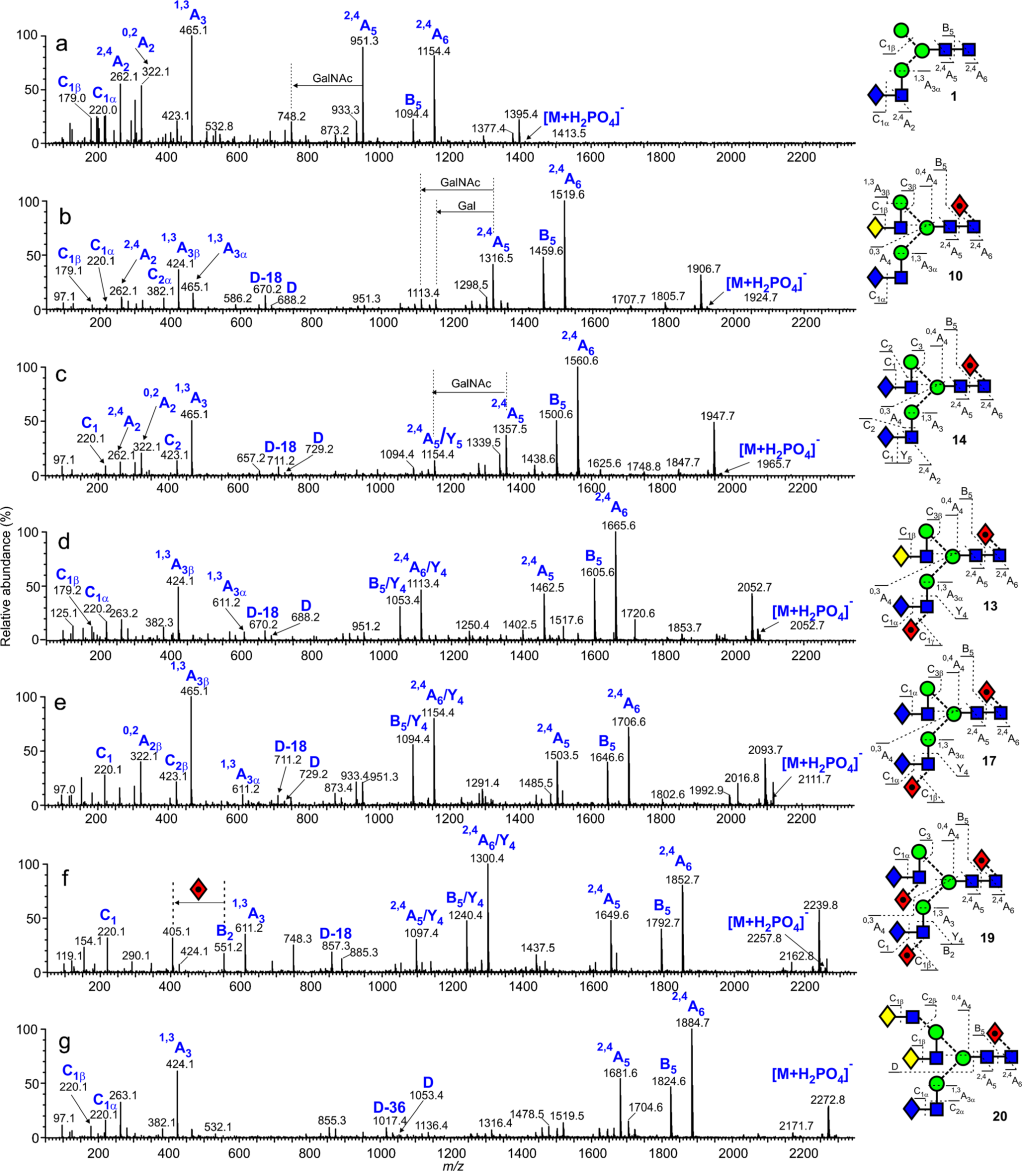
Negative ion CID spectra of (**a**) glycan **1** from hFSH, (**b**) the biantennary glycan **10** from gp41, (**c**) the biantennary glycan **14** with two GalNAc-capped antennae from gp41, (**d**) the difucosylated biantennary glycan **17** from gp41, (**e**) the difucosylated biantennary glycan **17** from gp41, (f) the trifucosylated biantennary glycan **19** from gp41, and (**f**) the triantennary glycan **20** from gp41. Symbols for the glycans are as described in the legend to Fig. 1

Figure 3 shows a selection of the negative ion CID spectra of several of the glycans whose antennae terminate in GalNAc on both gp120 and gp41. All but that from glycan **1** (panel a) were recorded with the TWIMS instrument whereas that from glycan **1** was obtained with a standard Q-TOF mass spectrometer and, thus, some of the minor ions may be from contaminating compounds. The use of ion mobility to record the other spectra enabled any contaminating ions to be removed.

The major ion identifying the GalNAc-GlcNAc-containing antennae was the ^1,3^A_3_ ion at *m*/*z* 465.1. This was the major ion in the MS/MS spectrum of glycan **1** (Fig. 3a). The C_1α_ ion at *m*/*z* 220.1 and the ^2,4^A_2_ ion at *m*/*z* 262.1 further characterized the structure of the antenna. The core di-*N*-acetyl-chitobiose region was defined by the ^2,4^A_6_, B_5_, and ^2,4^A_5_ ions at *m*/*z* 1154.3, 1094.3, and 951.3 respectively. This pattern of three ions was repeated in the spectra of all glycans in Fig. 3 and enabled the location of core fucose to be confirmed (see below). Loss of the GalNAc residue from the ^2,4^A_5_ ion to give *m*/*z* 748.2 further confirmed the presence of GalNAc at the non-reducing terminus of glycan **1**. Location of the GalNAc-GlcNAc chain on the mannose residue attached to the 3-position of the core-branching mannose was made by comparison with the spectra of the other glycans, all of which had the GalNAc-GlcNAc located at this position. This conclusion was supported by the absence of a D-18 fragment ion at *m*/*z* 711, a prominent fragment in the spectra of the glycans containing GalNAc-GlcNAc as the 6-antenna.

The *m*/*z* 1924.7 ion ([M+H_2_PO_4_]^−^) in the glycan profiles was produced by the biantennary glycan **10** containing one GalNAc residue. Its CID spectrum is shown in Fig. 3b. Two ^1,3^A_3_ ions were present; the first at *m*/*z* 424.1 contained Gal-GlcNAc (^1,3^A_3β_) and the second at *m*/*z* 465.1 contained the GalNAc-GlcNAc chain (^1,3^A_3α_). Two C_1_ fragments at *m*/*z* 179.1 (C_1β_) and 220.1 (C_1α_) were consistent with the two antennae terminating with galactose and GalNAc respectively. Ions generated from the loss of both galactose (*m*/*z* 1154.4) and GalNAc (*m*/*z* 1113.4) respectively from the ^2,4^A_5_ ion confirmed the terminal sugar residues at the non-reducing terminus. A common feature of the fragmentation of negative ions from these glycans is the formation of an ion, termed ion D, formed by formal loss of the *N,N*-di-acetyl-chitobiose (GlcNAc-GlcNAc) core and the antenna linked to the 3-position of the branching mannose. It is accompanied by a second ion formed by loss of H_2_O and termed D-18 and two cross-ring fragments of the branching mannose, ^0.3^A and ^0,4^A, the latter ions being particularly prominent in the spectra of the high-mannose glycans. The masses of the D and D-18 ions in the spectrum of the biantennary glycan, **10**, were *m*/*z* 688.2 and 670.2 respectively showing that the Gal-GlcNAc antenna was located at the 6-position of the branching mannose leaving the GalNAc-GlcNAc antenna on position 3. Location of the GalNAc residue specifically to the 3-antenna of a biantennary glycan has previously been reported from human pituitary follicle-stimulating hormone [15]. The presence of GalNAc in the 6-antenna would have caused a shift of the D and D-18 ions by 41 units to *m*/*z* 728.2 and 711.2; no such ions were present. Location of the fucose residue to the 6-position of the reducing terminal GlcNAc residue was revealed by the masses of the ^2,4^A_6_, B_5_, and ^2,4^A_5_ ions (*m*/*z* 1519.5, 1459.5, and 1316.5 respectively), all of which lacked the fucose residue. In other respects, the spectrum paralleled that of a reference sample of the biantennary glycan with two galactose residues (**27**). The CID spectrum of the biantennary glycan **7** lacking the fucose residue, from the FSH sample, was virtually identical.

A fucosylated biantennary glycan (Hex_3_HexNAc_6_Fuc_1_) with both antennae terminating in GalNAc (**14**, *m*/*z* 1965.7) was also present in the gp41 and FSH samples. Its CID spectrum (Fig. 3c) exhibited no ion at *m*/*z* 424.1 that would be expected from a glycan with Gal-GlcNAc-antennae; instead, this ion was replaced by the prominent ^1.3^A_3_ ion at *m*/*z* 465.1. Absence of the C_1_ ion at *m*/*z* 179.1 and the presence of *m*/*z* 220.1 confirmed that both antennae terminated with GalNAc. The C_2_ ion appeared at *m*/*z* 423.1 confirming the presence of the GalNAc-GlcNAc moiety. D and D-18 ions at *m*/*z* 728.2 and 711.2 confirmed inclusion of GalNAc in the 6-antenna. Jin et al. [19] have reported the spectrum of the [M-2H]^2−^ ion from the corresponding glycan lacking fucose and have also observed the ^2,4^A_2_ (*m*/*z* 262.1), ^0.2^A_2_ (*m*/*z* 321.1), C_2_ (*m*/*z* 423.1), and ^1,3^A_3_ (*m*/*z* 465.1) ions. The singly charged ^2,4^A_5_ ion at *m*/*z* 1356.4 was of low abundance but the doubly charged version of this ion at *m*/*z* 678.3 was the base peak. The ^2,4^A_6_ ion, if present, (not labelled) was of very low abundance or absent.

Biantennary glycans were also present with a fucose residue on the antennae, additional to that on the reducing terminal GlcNAc residue. Thus, the spectrum of the difucosylated glycan **13** (*m*/*z* 2070.7) containing one GalNAc residue is shown in Fig. 3d. The D and D-18 ions appeared at *m*/*z* 688.2 and 670.2 confirming that the 6-antenna terminated in galactose and that the GalNAc was in the 3-position as in the glycans whose spectra have already been discussed. The presence of fucose on the GalNAc-containing antenna caused a shift of the GalNAc-containing ^1,3^A_3_ ion from *m*/*z* 465.1 to *m*/*z* 611.2. No corresponding shift was noted for the galactose-containing ion at *m*/*z* 424.1 showing the absence of fucose on the galactose-containing antenna. The C_1α_ ion at *m*/*z* 220.1 showed that the fucose was not located on the terminal GalNAc residue. Work by other investigators on the structure of *N*-glycans on glycoproteins expressed in HEK 293 cells has also shown that fucose that is attached to an antennae is linked at the 3-position of the GlcNAc residue [20–22]. Furthermore, fucose substitution on galactose has been found to produce a prominent pair of ions at *m*/*z* 427.1 and 409.1 produced by an ^0,2^A_3_ cleavage followed by loss of H_2_O [42]. These ions and the corresponding predicted ones (*m*/*z* 468.1 and 450.1) from a GalNAc-containing antenna were absent, leaving little doubt that the attachment of fucose was to the 3-position of the GlcNAc residue. We have found fucose at this position in glycans from the human parotid gland (Gal-GlcNAc-antennae) [7] and in their spectra the abundance of the ions formed by loss of the fucosylated 3-antenna (GalNAc-(Fuc)GlcNAc) from the ^2,4^A_6_, B_5_, and ^2,4^A_5_ ions is considerably elevated compared with the spectra of corresponding compounds where there is no fucose attached to the GlcNAc residue [7]. The same phenomenon was noted in these GalNAc-containing spectra (*m*/*z* 1113.4, 1053.4, and 951.3 in spectrum 3d).

Glycan **17** (*m*/*z* 2111.7) with two GalNAc and two fucose residues gave a spectrum (Fig. 3e) with similar characteristics again indicating fucose substitution at the 3-position of the GlcNAc of the 3-antenna, The D and D-18 ions appeared at *m*/*z* 729.2 and 711.2 confirming that the 6-antenna terminated in GalNAc and showing no fucose substitution on this antenna. Again, loss of GalNAc-(Fuc)GlcNAc from the ^2,4^A_6_, B_5_, and ^2,4^A_5_ ions produced major fragments at *m*/*z* 1154.4, 1094.4, and 951.3 respectively.

A fucosylated GalNAc-containing glycan (HexNAc_6_Hex_3_Fuc_3_) containing three fucose residues gave the [M+H_2_PO_4_]^−^ ion at *m*/*z* 2257.8. Its CID spectrum (Fig. 3f) contained no ^1,3^A_3_ ion at *m*/*z* 465.1 (unsubstituted antenna) but a prominent one at *m*/*z* 611.2 indicating fucose linked to an antenna. No di-fucosylated equivalent (*m/z* 757.3) was detected showing that each antenna contained a single fucose residue. Again the C_1_ ion at *m*/*z* 220.1 (no fucose) and the absence of the ^0,2^A_3_ ions at *m*/*z* 468.2 and 450.2 showed that the fucose residues were located on the GlcNAc residues to give structure **19**.

The [M+H_2_PO_4_]^−^ ion at *m*/*z* 2289.8 resolved to a composition of Hex_5_HexNAc_6_Fuc_1_. Its CID spectrum (Fig. 3g) contained ^1,3^A_3_ ions at *m*/*z* 424.1 and 465.1 showing the presence of Gal-GlcNAc and GalNAc-GlcNAc-antenna. D, D-18, and D-36 ions at *m*/*z* 1053.4, 1035.4, and 1017.4 respectively were consistent with a triantennary glycan containing an unsubstituted branched 6-antenna (two Gal-GlcNAc chains) [43] leaving GalNAc-GlcNAc as the structure of the 3-antenna as found in the biantennary glycan **10**. Location of the fucose residue to the core was indicated by the masses of the ^2,4^A_6_, B_5_, and ^2,4^A_5_ ions at *m*/*z* 1852.7, 1792.7, and 1649.6 respectively. This glycan was, therefore, identified as **20**. The weak CID spectrum (not shown) of a very minor glycan containing two fucose residues (*m*/*z* 2435.8, (HexNAc_6_Hex_5_Fuc_2_)) contained D-36 (*m*/*z* 1017.4) and ^1,3^A_3_ (*m*/*z* 611.2) ions supporting the structure **25**.

Small amounts of different hybrid glycans containing GalNAc were also detected from both gp41 and gp120, but these were isobaric with other major glycans and individual, clean spectra were not obtained. However, by comparing the spectra with those of reference spectra (Dextra Laboratories) of the major contaminating glycans, the ions belonging to the hybrid glycans could be identified. In some cases, there was a difference in drift times allowing the relevant fragment ions to be isolated. Thus, the hybrid glycan **6** (HexNAc_4_Hex_5_, *m*/*z* 1737.6), isobaric with the biantennary glycan **7**, was identified by the presence of the usual D, D-18, ^0,3^A_3_, and ^0,4^A_3_ ions (*m*/*z* 647.2, 629.2, 575.2, and 545.2 respectively) showing a mannose-containing 6-antenna as in the CID spectrum of the high-mannose glycan, Man_5_GlcNAc_2_, which has the same mannose residues in the 6-antenna [6] and the GalNAc-GlcNAc-containing ^1,3^A_3_ ion at *m*/*z* 465.1. The sets of fragment ions from the two compounds showed slight separation. The cross section measured in the MS spectrum for the mixture of glycans **6** and **7** was 392.2 Å^2^. Extracted fragment ATDs of the ^1,3^A_3_ ions (*m*/*z* 424.1 for **7** and 465.2 for **6**) in the CID spectrum, diagnostic for the two compounds, allowed further measurements of the cross sections of these two compounds to be made. These measurements gave values of 393.8 Å^2^ for the biantennary glycan **7** and 391.3 Å^2^ for the hybrid glycan **6**.

The fucosylated analogues of compound **6** (*m*/*z* 1883.6) are isobaric with several other potential glycans including the fucosylated biantennary glycan **28**. In addition to the ions characterizing this latter glycan in Fig. 3a, the spectrum contained very minor D, D-18, ^0,3^A_3_, and ^0,4^A_3_ ions at *m*/*z* 647.2, 629.2, 575.2, and 545.2 characterizing the mannose residues of the 6-antenna of compounds such as **8** and **9**. This conclusion was consistent with the presence of minor ^2,4^A_6_, B_5_, and ^2,4^A_5_ ions at *m*/*z* 1624.6, 1564.6, and 1421.6 respectively whose ion mobility drift times aligned with those of *m*/*z* 611.2 but differed from those of the glycans with a fucosylated core GlcNAc. These observations provided evidence for the presence of glycan **9**. Although the core-fucosylated glycan **8** was probably present, its presence could not be confirmed. The ^1,3^A_3_ ion at *m*/*z* 465.1 diagnostic for GalNAc-GlcNAc was of very low relative abundance and the ^2,4^A_6_, B_5_, and ^2,4^A_5_ ions were isobaric with those from the biantennary glycan **28** and showed no mobility separation.

Analogues of all of these GalNAc-containing hybrid glycans containing one fewer mannose residue in the 6-antenna and with zero (**2**, *m*/*z* 1575.5), one (**3** and **4**, *m*/*z* 1721.6, Fig. 3b), and two (**5**, *m*/*z* 1867.6) fucose residues were detected by the presence of the appropriate diagnostic fragment ions as discussed earlier.

## Sulfated glycans

**Fig. 4:**
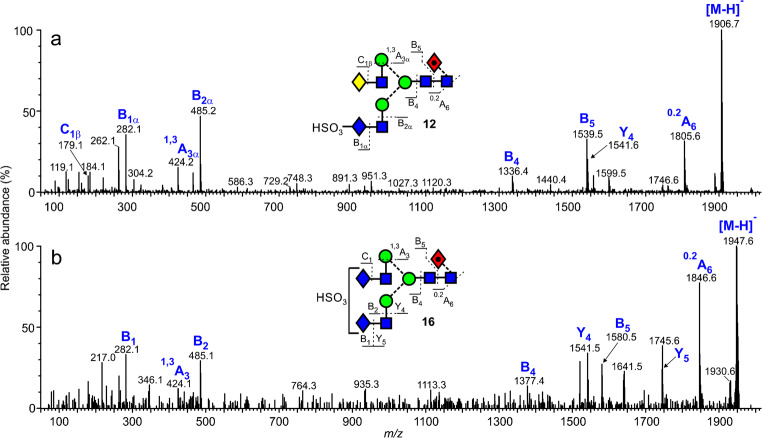
Negative ion CID spectra of the sulfated glycans **12** and **16**

Released *N*-glycans whose antennae terminate in GalNAc often contain a 4-linked sulfate residue [17, 22, 44]. Several such glycans were found in the mixture of glycans from gp120 and gp41. Thus, Fig. 4a shows the spectrum of the glycan **12** (*m*/*z* 1906.6, [M-H]^−^ ion) of composition HexNAc_5_Hex_4_Fuc_1_-SO_3_H. The spectrum contained prominent fragment ions at *m*/*z* 282.1 (B_1α_, HSO_3_-GalNAc) and 485.2 (B_2α_, HSO_3_-GalNAc-GlcNAc) as found earlier for sulfated glycans of this type [9]. Ions at the high-mass end of the spectrum included the ^0,2^A_6_ ion at *m*/*z* 1805.6, an ion frequently present in the negative ion spectra of acidic glycans. Other ions were mainly B fragments with the B_5_ fragment consistent with the presence of fucose on the core GlcNAc. Glycan **16** had a similar structure but with an additional GalNAc residue in place of the galactose. Its spectrum (Fig. 4b) was similar to that of glycan **12**. Additional Y ions at *m*/*z* 1745.6 and 1541.6 arose from loss of GalNAc and GalNAc-GlcNAc respectively from the antenna not containing the sulfate group. From the rather weak spectrum, it was not possible to determine which antenna carried the sulfate group. The sulfated glycans **18** (*m*/*z* 2093.7) and **24** (*m*/*z* 2271.8) were identified similarly.

### Sialylated glycans

Most of the above neutral glycans produced sialylated analogues. All were sensitive to sialidase, giving the corresponding neutral glycans. Monosialylated glycans (**11**, **15**, **21**, and **26**) gave [M-H]^−^ ions. Doubly and triply charged negative deprotonated ions were formed by the more highly sialylated glycans. Both identified compounds (**22** and **23**) were sialylated derivatives of the neutral glycans discussed above. The CID spectra of all sialylated glycans contained a prominent B_1_ fragment at *m*/*z* 290.1 defining the Neu5Ac group and the absence of a fragment ion at *m*/*z* 306.2 in the samples from the HIV glycans showed that all sialic acid groups were α2→3-linked [9, 45]. α2→6-linked sialic acid was detected in the FSH samples [16].

## Conclusions

Gp120 and gp41 from BG505 SOSIP.664 trimers produced in HEK 293T cells were found to contain a complex mixture of *N*-glycans including 25 hybrid and complex glycans whose antennae terminated in GalNAc. They were easily identified by the presence of an abundant ^1,3^A cross-ring fragment at *m*/*z* 465.1. Fucose substitution on an antenna produced a shift of this ion to *m*/*z* 611.2. In other respects, these compounds fragmented in a similar manner to other hybrid and complex glycans reported earlier. D and D-18 ions, diagnostic of the composition of the 6-antenna, were present and could be used to define the branching pattern of the triantennary glycans. The masses of these ions showed that the location of the GalNAc residue in glycans with one GalNAc was invariably on the 3-antenna, a property that would be difficult to determine by traditional positive ion fragmentation methods. However, recent methods such as electronic excitation dissociation (EED) do appear to produce ions that allow this structural feature to be determined directly [46]. The current paper, therefore, provides a further illustration of how ion mobility and negative ion CID, combined with exoglycosidase digestions to determine the nature of the monosaccharide constituents, can be used to provide detailed structures of these compounds without the need for extensive fractionation or derivatization.

## Abbreviations

ATD: Arrival time distribution
CCS: Collisional cross section
CID: Collision-induced dissociation
EED: Electronic excitation dissociation
ESI: Electrospray
Fuc: Fucose
Gal: Galactose
GalNAc: *N*-Acetylgalactosamine
Glc: Glucose
GlcNAc: *N*-Acetylglucosamine
HEK: Human embryonic kidney
Hex: Hexose
HexNAc: *N*-Acetylhexosamine
hFSH: Human pituitary follicle-stimulating hormone
HIV: Human immunodeficiency virus
MS: Mass spectrometry
Neu5Ac: *N*-Acetylneuraminic acid (sialic acid)
PNGase F: Peptide *N*-glycosidase F
Q: Quadrupole
RF: Radio frequency
TOF: Time-of-flight
TWIMS: Travelling wave ion mobility mass spectrometry
